# Phytochemical Profile and Biological Activities of Different Extracts of Three Parts of *Paliurus spina-christi*: A Linkage between Structure and Ability

**DOI:** 10.3390/antiox12020255

**Published:** 2023-01-23

**Authors:** Gokhan Zengin, Álvaro Fernández-Ochoa, María de la Luz Cádiz-Gurrea, Francisco Javier Leyva-Jiménez, Antonio Segura-Carretero, Fevzi Elbasan, Evren Yildiztugay, Sumira Malik, Asaad Khalid, Ashraf N. Abdalla, Mohamad Fawzi Mahomoodally

**Affiliations:** 1 Department of Biology, Faculty of Science, Selcuk University, Selcuklu, Konya 42130, Turkey; 2 Department of Analytical Chemistry, Faculty of Sciences, University of Granada, Fuentenueva s/n, 18071 Granada, Spain; 3 Department of Analytical Chemistry and Food Science and Technology, University of Castilla-La Mancha, Ronda de Calatrava 7, 13071 Ciudad Real, Spain; 4 Regional Institute for Applied Scientific Research (IRICA), Area of Food Science, University of Castilla-La Mancha, Avenida Camilo Jose Cela, 10, 13071 Ciudad Real, Spain; 5 Department of Biotechnology, Faculty of Science, Selcuk University, Selcuklu, Konya 42130, Turkey; 6 Amity Institute of Biotechnology, Amity University Jharkhand, Ranchi 834001, India; 7 Substance Abuse and Toxicology Research Center, Jazan University, P.O. Box 114, Jazan 45142, Saudi Arabia; 8 Medicinal and Aromatic Plants and Traditional Medicine Research Institute, National Center for Research, Khartoum P.O. Box 2404, Sudan; 9 Department of Pharmacology and Toxicology, College of Pharmacy, Umm Al-Qura University, Makkah 21955, Saudi Arabia; 10 Department of Health Sciences, Faculty of Medicine and Health Sciences, University of Mauritius, Réduit 230, Mauritius; 11 Center for Transdisciplinary Research, Department of Pharmacology, Saveetha Dental College, Saveetha Institute of Medical and Technical Science, Chennai 600077, India; 12 Centre of Excellence for Pharmaceutical Sciences (Pharmacen), North West University, Potchefstroom 2520, South Africa

**Keywords:** *P. spina-christi*, phytochemicals, antioxidant, enzyme inhibition

## Abstract

*Paliurus spina-christi* Mill., a member of the Rhamnaceae family, is a traditionally used medicinal plant in the management of a panoply of human ailments. The current research focused on its phytochemical profile and biological properties evaluated by its antioxidant and enzyme inhibitory properties. The methanol extract was found to be the most effective antioxidant as evidenced by its DPPH and ABTS scavenging activities, cupric and ferric reducing power (CUPRAC and FRAP), and high activity in phosphomolybdenum (PBD) assay, and also displayed the highest anti-tyrosinase activity. The n-hexane extract was the most effective AChE inhibitor (8.89 ± 0.08 mg GALAE/g) followed by the methanol (8.64 ± 0.01 mg GALAE/g) while the latter showed the highest BChE inhibition (2.50 ± 0.05 mg GALAE/g). Among the different solvent extracts of the stem, the methanolic extract showed highest antioxidant activity in the following assays: DPPH (909.88 ± 4.25 mg TE/g), ABTS (3358.33 ± 51.14 mg TE/g), CUPRAC (781.88 ± 16.37 mg TE/g), FRAP (996.70 ± 47.28 mg TE/g), and PBD (4.96 ± 0.26 mmol TE/g), while the dichloromethane extract showed the highest MCA (28.80 ± 0.32 mg EDTAE/g). The methanol extracts revealed the highest TPC and TFC among the different solvents used, and as for plant part, the stem extracts had the highest TPC ranging from 22.36 ± 0.26 to 121.78 ± 1.41 (mg GAE/g), while the leaf extracts showed the highest TFC ranging from 8.43 ± 0.03 to 75.36 ± 0.92 (mg RE/g). Our findings tend to provide additional scientific evidence on the biological and chemical activities of *P. spina-christi*, which may serve as a source of naturally occurring bioactive chemicals with potential biomedical applications.

## 1. Introduction

Noncommunicable diseases (NCDs), also known as chronic diseases, occur as a result of a combination of physiological, genetic, environmental, and socioeconomic factors. These disorders have low progress and long duration. It is estimated that one-third of the death rate caused by NCDs can be decreased by 2030 by reducing risk factors such as intake of tobacco and alcohol, lack of proper diet, lack of physical exercise, as well as ensuring proper disease diagnosis and treatment [1].

Although adjustments in daily lifestyle and diet are still considered important, studies suggest that increased dietary intake of antioxidants plays a crucial role in the prevention of NCDs, especially when taken with a normal diet. Several findings have shed light on the positive impact of phytochemicals on humans, among which polyphenols are one of the most abundant and nutritionally important phytochemicals. There are more than 8000 phenolic structures presently known and more than 500 are found in food plants and regarded as dietary polyphenols [2].

In addition to the use of antioxidants, enzyme inhibition is another key approach in targeting NCDs. Despite the life-essential nature of enzyme catalysis, there exists conditions in which changes in enzyme activity can lead to diseases. For infectious diseases, specific enzymes of the invading organism which are necessary for its survival or replication can be targeted for drug therapy. For chronic diseases, changes in genetic and/or environmental conditions can lead to expression-based, mutation-based or post-translational modification-based gain-of-function alterations in enzyme activity. Consequently, molecules that cause inhibition of the affected enzyme could be useful therapeutic agents [3].

Turkey has a rich flora of plants of which one third is endemic to the country. The traditional use of medicinal plants is a common practice and according to ethnobotanical field studies, 1546 species are recorded as medicinal plants in Turkey [4]. Compared to other European countries, the rate of endemism of plant species in Turkey is relatively high. East Anatolia has a rich flora because of its variable climate and great number of ecological zones. This diversity in flora provides a rich source of medicinal plants, accounting for the accumulation of remarkable medicinal indigenous knowledge in the region [5].

*Paliurus spina-christi* Mill. is a member of the Rhamnaceae family which is used traditionally to manage several ailments. It is used as a diuretic and against diarrhoea and rheumatism [6]. The fruits are used as anti-inflammatory agents against kidney stones, chest and eye infections, while the leaves are used externally against boil inflammation. The plant is also used for the treatment of diabetes mellitus in Turkey. There are several species from the *Rhamnaceae* family and about five species from the *Paliurus* genus, of these, only *P. spina-christi* Mill. is present in Turkey’s flora [7].

Several studies have proved the biological properties of this plant including antioxidant [8,9,10] and antidiabetic [7] properties. The ethanolic extracts of different plant parts (leaf, flower, fruit, bark and root) showed antibacterial properties against gram-positive bacteria including *Staphylococcus aureus*, *Streptococcus faecalis*, and *Micrococcus luteus* [6]. In the study by [8], among the tested microorganisms, the ethanolic extract of the fruit was more effective against *Pseudomonas aeruginosa*, *Klebsiella pneumoniae*, and *Candida albicans*. The antifungal activity of the methanol extract was also evidenced against *Trichophyton mentagrophytes* [11]. Furthermore, the anti-inflammatory activity of the fruit, leaf and branch extracts was studied by Şen [9]. The ethyl acetate and ethanol extracts of the leaves were found to have the best anti-inflammatory activity in the lipoxygenase inhibition assay [9]. In addition, the antigenotoxic properties of the fruits and their active compounds such as catechins, gallocatechin, and rutin were also evidenced, which explained its protective effects in oxidative DNA damage [12]. In addition, Ferdi, et al. [13] studied the apoptotic effect of the leaf and flower extracts against breast cancer cells. Significant cytotoxic effects were observed after 72 h treatment in MCF-7 cells but not in MDA-MB-231 cells. Both leaf and flower extracts induced apoptotic cell death in MCF-7 cells.

Although there have been a number of in vitro pharmacological studies, the in-depth biological properties of *P. spina-christi* are yet to be explored. Consequently, this study aims to establish its phytochemical profile, antioxidant activity, and enzyme inhibitory potential against key enzymes linked to chronic diseases.

## 2. Materials and Methods

### 2.1. Plant Materials and Extraction

The fruits, leaves and stems of the plants (*Paliurus spina-christi*) were collected in Isparta (Yazılı Kanyon National Park), Turkey, in the summer season of 2020. The plant was identified by one botanist co-author (Dr. Evren Yildiztugay, Selcuk University). Voucher specimens (EY-3132) were deposited at the herbarium in Selcuk University.

The plant samples were randomly collected from twenty plants of the same population in a given area. In the preparation of plant extracts from each plant part, we used four solvents (n-hexane, ethyl acetate, dichloromethane, methanol, and water) to extract compounds with different polarities. The maceration technique was selected for organic solvents and for this purpose, plant materials (10 g) were stirred with the 200 mL of methanol for 24 h at room temperature. After that, the mixtures were filtered using Whatman filter paper and the solvents were removed using a rotary-evaporator. Regarding a water extract, the extract was prepared as a traditional infusion and the plant materials (10 g) were kept in the boiled water (200 mL) for 15 min. Then, the mixture was filtered and lyophilized for 48 h. All extracts were stored at 4 °C until analysis.

### 2.2. Chemical Reagents

All chemicals were of LC-MS grade. Acetic acid and acetonitrile for UPLC were purchased from Fluka (Sigma-Aldrich, Steinheim, Germany) and Lab-Scan (Gliwice, Sowinskiego, Poland), respectively. For solutions, ultrapure water was obtained with a Milli-Q system Millipore (Bedford, MA, USA), and methanol was purchased from Honeywell (Wabash, IN, USA).

### 2.3. Total Phenolic and Flavonoid Content

The total phenolic content (TPC) and total flavonoid content (TFC) were determined spectrophotometrically as described in [14]. Data were expressed as mg gallic acid equivalents (GAE)/g extract in TPC and mg rutin equivalents (RE)/g extract in TFC. All measurements were performed in triplicate.

### 2.4. UPLC-ESI-QTOF-MS Conditions

Paliurus extracts were redissolved at 5 mg/mL in water or methanol depending the extraction conditions and then were filtered by 0.22 µm. The separation process was carried out by an ACQUITY UPLC H-Class System (Waters Corp., Milford, MA, USA) with a reversed-phase column (ACQUITY UPLC BEH Shield RP18, 130Å, 1.7 µm, 2.1 mm × 150 mm). The flow rate was 0.7 mL/min and the volume injection of each sample was 10 μL. The mobile phases were A: acidified water (0.5% acetic acid, *v*/*v*) and B: acetonitrile. The following multi-step linear gradient was used for achieving an efficient separation: 0.00 min, 99% A; 2.33 min, 99% A; 4.37 min, 93% A; 8.11 min, 86% A; 12.19 min, 76% A; 15.99 min, 60% A; 18.31 min, 2% A; 21.03 min, 2% A; 22.39 min, 99% A and 25.00 99% A.

The UPLC was coupled to an electrospray quadrupole time-of-flight mass spectrometer (ESI-QTOF-MS) Synapt G2 (Waters Corp., Milford, MA, USA) working in negative-ion mode. The *m*/*z* range was from 50 to 1200 *m*/*z*. The MS acquisition was based on two parallel scan functions switching between them continuously. Leu-enkephalin was injected for mass calibration continuously. Other MS parameters were as follows: source temperature 100 °C; scan duration 0.1 s; resolution 20,000 FWHM; desolvation temperature 500 °C; desolvation gas flow 700 L/h; capillary voltage 2.2 kV; cone voltage 30 V; cone gas flow 50 L/h. Finally, the acquired data were processed using MZmine 2.53 open-source software [15] and Sirius 4.4.29 [16].

### 2.5. Antioxidant Assays

The DPPH radical scavenging, ABTS radical scavenging, cupric ion reducing antioxidant capacity (CUPRAC), ferric ion reducing antioxidant power (FRAP), metal chelating activity (MCA), and phosphomolybdenum (PBD) were determined as presented in [14,17]. The activity data were expressed as mg Trolox equivalents (TE)/g extract in DPPH, ABTS, CUPRAC, and FRAP assay; mg EDTA equivalents (EDTAE)/g extract in the MCA assay, and mmol TE/g extract in the PBD assay.

### 2.6. Enzyme Inhibitory Assays

AChE, BChE, tyrosinase, amylase, and glucosidase inhibition were determined as presented in [14,17]. The activity data were expressed as mg galanthamine equivalents (GALAE)/g extract in the AChE and BChE assays, mg kojic acid equivalents (KAE)/g extract in tyrosinase assay, and mmol acarbose equivalents (ACAE)/g extract in amylase and glucosidase assays.

### 2.7. Statistical Analysis

Data are presented as mean ± standard deviation of the number (n = 3) of replicates. One-way analysis of variance with Tukey’s post-hoc test was conducted; *p* < 0.05 was considered statistically significant. The statistical evaluation was performed using Graphpad version 9.0.

## 3. Results

### 3.1. Phytochemical Analysis

The present study revealed a difference in total phenolic content (TPC) and total flavonoid content (TFC) among different solvent extracts of *P. spina-christi* (Table S1). With regard to the stem, TPC of the various extracts ranged from 22.36 ± 0.26 to 121.78 ± 1.41 mg GAE/g. The order of solvents with highest TPC was methanol > water > ethyl acetate > dichloromethane > n-hexane. The TFC were in the range 0.83 ± 0.07 to 11.25 ± 0.26 mg RE/g in the order methanol > dichloromethane > ethyl acetate > water > n-hexane. The methanol extract of the fruits also showed the highest TPC (75.91 ± 0.58 mg GAE/g) followed by the water, ethyl acetate, n-hexane, and then dichloromethane extract. Likewise, TFC were in the order methanol > water > ethyl acetate > dichloromethane > n-hexane in the range 0.14 ± 0.03 to 17.55 ± 0.09 mg RE/g. As for the leaf, the methanol extract also displayed the highest TPC and TFC of 94.64 ± 2.12 mg GAE/g and 75.36 ± 0.92 mg RE/g, respectively. In general, TPC of the solvent extracts of the leaf was in the order methanol > water > ethyl acetate > n-hexane > dichloromethane while TFC was in the order methanol > water > dichloromethane > ethyl acetate > n-hexane. Overall, the methanol extracts revealed the highest TPC and TFC among the different solvents used.

Some studies have been previously conducted on the phytochemical profile of this plant. The TPC amount of fruit extract was previously found to be 22.10 ± 0.09 mg GAE/g dry plant, while TFC was 8.29 ± 0.07 mg quercetin equivalent/g dry plant [10]. The flavonoid spectrum in different parts of the plant was determined using reversed-phase HPLC. Quercetin 3-*O*-rhamnoglucoside 7-*O*-rhamnoside and rutin were revealed to be the main flavonoid components in the leaves, flowers, and fruits [6]. LC-MS/MS analysis of the fruit also revealed that malic acid (283 μg/g extract) and rutin (233 μg/g extract) are the highest among 22 phenolic compounds [10]. In another study by [9], the highest TPC was found in the ethyl acetate extract of branches of *P. spina-christi* (286.6 mg/g) while the TPC of other extracts ranged between 2.44 and 216.2 mg GAE per g extract. In the study by [7], phytochemical analysis was carried out with optimized and validated LC-MS/MS method on *P. spina-christi* fruits. The mineral content was also determined using ICP-OES analysis. A total of 31 different phenolic compounds were identified. The major compounds were rutin (98,753 ± 24 μg), catechin (58,695 ± 13 μg), hesperidin (47,445 ± 16 μg), quinic acid (382,780 ± 14. μg) and malic acid (17,537 ± 2 μg). As for minerals, sodium, calcium, magnesium and phosphorus were found at macro level, while Zn and Cr^3+^ were found at trace level.

Furthermore, Zor et al. [12] found that the major identified compounds of the fruits were (±) catechins and gallocatechin from the ethyl acetate fraction and rutin from the n-butanol fraction. Their chemical structures were identified by 1 H-NMR, 13C-NMR, HMBC, and HMQC techniques. Further phytochemical screening by Ferdi et al. [13] showed that the leaf extract contains pyrrolidine, 2-decenal, 2-undecanal, phytol, oleic acid, oleamide, squalane, vitamin E, and gamma-sitosterol and also found that the flower extracts possess pyrrolidine, 2-decenal, 2-undecenal, oleic acid, lupeol, and gamma-sitosterol. In addition, high performance liquid chromatography analysis of the methanolic extract of the stem revealed the presence of gallic acid, cafetric acid, syringic acid and epichotichen, while gas chromatography revealed a wide range of sugar compounds, phenols, alkaloids, and esters [11].

### 3.2. Characterization of Bioactive Compounds from Paliurus spina-christi Water and Methanolic Extracts from Twigs, Flowers and Leaves by UPLC—ESI-QTOF-MS

Following the described analytical method, all extracts have been analyzed resulting in a total of 146 detected compounds. Figure S1 shows the base peak chromatograms performed for the analyzed *Paliurus* extracts (water and methanol) from different parts of the plant. In addition, Table 1 summarizes all information about detected compounds such as retention time, *m*/*z* ratio, error in ppm, molecular formula, name of each proposed compound and area of each compound in the different extracts. Regarding twigs, 39 compounds were annotated in methanol extract and 67 in water extract. The most abundant signals in both extracts were quercetin and epigallocatechin derivatives. When fruit extracts were analyzed, 24 and 20 compounds were detected in methanol and water extracts, respectively, with sugar and quercetin derivatives being the most abundant compounds in their profiles. In leaf extracts, kaempferol derivatives presented the major contribution to the methanolic extract where 54 compounds were detected in total. However, water leaf extract showed 78 compounds, with flavonoids, such as kaempferol and quercetin derivatives, the most abundant ones.

### 3.3. Antioxidant Activity

Oxidative stress plays a key role in the development of age-related diseases such as arthritis, diabetes, dementia, cancer, cardiovascular diseases, osteoporosis, and metabolic syndromes. Reactive oxygen species are normally generated within the biological system for modulation of cellular activities such as cell survival, stressor responses, and inflammation. However, a high level of reactive oxygen species can cause oxidative stress by disrupting the balance of antioxidant and prooxidant levels. Current research evidences have revealed that natural compounds with antioxidant properties can lower oxidative stress and improve immune function [18]. In the present study, the DPPH and ABTS scavenging properties, FRAP and CUPRAC reducing power, and other assays such as phosphomolybdenum (PBD) and metal chelating (MCA) were carried out to assess the antioxidant potential of the different *P. spina-christi* solvent extracts.

Among the different solvent extracts of the stem, the methanolic extract showed highest antioxidant (Table 2) activity in the following assays: DPPH (909.88 ± 4.25 mg TE/g), ABTS (3358.33 ± 51.14 mg TE/g), CUPRAC (781.88 ± 16.37 mg TE/g), FRAP (996.70 ± 47.28 mg TE/g), and PBD (4.96 ± 0.26 mmol TE/g) while the dichloromethane extract showed the highest MCA (28.80 ± 0.32 mg EDTAE/g). The water extract also showed high antioxidant potential but lower effect compared to the methanol extract (DPPH: 547.54 ± 25.39 mg TE/g, ABTS: 1926.18 ± 34.63 mg TE/g, CUPRAC: 506.98 ± 2.54 mg TE/g, FRAP: 688.38 ± 3.43 mg TE/g, PBD: 2.73 ± 0.05 mmol TE/g) but slightly higher MCA than methanol extract (9.65 ± 0.35 mg EDTAE/g). As for the fruit, the methanol extract also exhibited the strongest antioxidant effect in DPPH and ABTS scavenging (245.59 ± 4.46 and 824.40 ± 17.11 mg TE/g, respectively) as well as reducing power in CUPRAC and FRAP (282.66 ± 11.38 and 292.94 ± 6.60 mg TE/g, respectively) while the water extract was most effective in MCA (21.80 ± 0.59 mg EDTAE/g) and PBD (1.80 ± 0.03 mmol TE/g). Likewise, for the leaf extract, the methanol extract was the most effective antioxidant, as observed in the different assays [DPPH (480.10 ± 7.80 mg TE/g), ABTS (1171.58 ± 25.83 mg TE/g), CUPRAC (506.98 ± 7.27 mg TE/g), FRAP (664.85 ± 0.49 mg TE/g), and PBD (2.76 ± 0.19 mmol TE/g)] except for MCA, in which the dichloromethane extract was most efficient (23.44 ± 0.03 mg EDTAE/g). The water extract also displayed high antioxidant power but less than that of the methanol extract.

The high antioxidant activity of the methanol and water extracts tend to tally with their TPC and TFC since these solvent extracts had higher content. Indeed, a number of studies have observed the positive correlation between TPC/TFC and the antioxidant efficacy of plant extracts [19,20,21]. It is to be noted that some previous studies have also observed the antioxidant potential of *P. spina-christi*. For instance, the study of Takım and Işık [10] revealed that the aqueous fruit extract displayed a high rate of DPPH and ABTS radical scavenging activity compared with ascorbic acid (*p* < 0.001), and higher effect in FRAP and CUPRAC assays compared with Trolox (*p* < 0.001, *p* < 0.05). The extract also significantly raised the enzyme activity of catalase and superoxide dismutase while reducing total glutathione and malondialdehyde in streptozotocin-induced diabetic rats.

Moreover, the antioxidant activity of the fruit, leaf, and branch extracts of *P. spina-christi* was also determined by Şen et al. [9]. All extracts of the branches, except hexane extract, exhibited high antioxidant activity against DPPH and ABTS radicals, especially the ethyl acetate and ethanol extracts displaying IC_50_ values of 15.54 and 22.06 µg/mL, respectively, in DPPH and ABTS assays. Additionally, Arslan and Kaya [8] studied the antioxidant properties of the water and ethanol extracts of the fruit and leaves. Using the CUPRAC method, the antioxidant effect of the extracts, tested at a concentration of 800 µg/mL, were found to be lower compared to those of standard antioxidants. At the same concentration, the DPPH radical scavenging activity of the ethanolic extract of the leaves (80.2% inhibition) was found to be stronger compared to Trolox (65.8% inhibition). As for ABTS assay, the radical scavenging activity of the fruit (96.2% inhibition) was found to be high and near to the effect of standard antioxidants.

### 3.4. Enzyme Inhibitory Activity

The enzyme inhibitory property of the extracts was evaluated against key enzymes related to chronic diseases [Acetylcholinesterase (AChE) and butyrylcholinesterase (BChE), tyrosinase, amylase, and glucosidase]. Inhibitors of AChE and BChE have found application as drugs developed for the treatment of Alzheimer’s disease which is a neurodegenerative disorder characterized by the loss of memory and consciousness. The inhibition of AChE and BChE is based on the theory that raising the availability of acetylcholine at acetylcholine receptors in the brain leads to better neuron to neuron transport, thereby improving cognitive function [22].

Among the stem extracts, the n-hexane extract was the most effective AChE inhibitor (8.89 ± 0.08 mg GALAE/g) followed by the methanol (8.64 ± 0.01 mg GALAE/g), while the latter showed the highest BChE inhibition (2.50 ± 0.05 mg GALAE/g). For fruit extracts, the n-hexane exhibited the greatest AChE inhibition (8.50 ± 0.32 mg GALAE/g) while the most efficient BChE inhibitor was the ethyl acetate (2.32 ± 0.13 mg GALAE/g) and dichloromethane (2.32 ± 0.10 mg GALAE/g) extracts followed by n-hexane (2.18 ± 0.05 mg GALAE/g). As for the leaf, the methanol and ethyl acetate displayed highest AChE inhibition (8.41 ± 0.30 and 8.37 ± 0.37 mg GALAE/g, respectively) while the dichloromethane showed highest BChE inhibition (1.98 ± 0.10 mg GALAE/g). It is important to highlight that although the n-hexane, ethyl acetate, and dichloromethane extracts were most effective in inhibiting cholinesterase enzyme activity, they were not rich in TPC and TFC. A possible explanation for this observation might be that a combination of some specific phenolic compounds, although present in low amounts, might be responsible for the observed cholinesterase inhibition. These specific compounds might also exhibit synergistic effect in combinations, producing an activity greater than the sum of the individual effects [23].

Among the different solvent extracts tested, the most efficient amylase inhibitors were the ethyl acetate and dichloromethane extracts (Stem: 0.55 ± 0.01 mmol ACAE/g; Fruit: 0.63 ± 0.01 and 0.55 ± 0.01 mmol ACAE/g, respectively; Leaf: 0.60 ± 0.01 mmol ACAE/g) (Table 3). As for glucosidase inhibition, among the stem extracts, the dichloromethane and methanol extracts were most effective (1.07 ± 0.21 and 1.06 ± 0.01 mmol ACAE/g, respectively). For the fruit and leaf, the water and methanol extracts showed highest inhibition in the range 0.98 ± 0.02 to 1.01 ± 0.02 mmol ACAE/g. The observed amylase and glucosidase inhibition can be supported by the in vivo findings of the study of Takım et al. [7] which investigated the antidiabetic effect of *P. spina-christi* fruits in diabetic rats induced by streptozotocin. When the plant extract groups were compared to the diabetic control group, it was observed that blood glucose and HbA1c levels were statistically reduced (*p* < 0.001) and that their diabetic conditions were regulated.

Plants are natural sources of various phytochemicals such as phenols, flavonoids, alkaloids, glycosides, lignins, and tannins. Phenols and flavonoids are the most common phytoconstituents responsible for antioxidant properties [24]. The presence of hydroxyl groups in the B-ring of flavonoids allows the donation of hydrogen atoms during free radical reactions. On top of that, phenolics are a good source of antioxidants acting via several mechanisms such as free radical-scavenging, hydrogen donation, singlet oxygen quenching, metal ion chelating, and also by acting as a substrate for radicals such as superoxide and hydroxyl [25].

Tyrosinase activity is involved in the synthesis content of melanin which is generally considered the perfect protection against UV damage. Exposure of skin to UV radiation or oxidative stress causes the melanocytes to release melanin, which accumulates in melanosomes, and then transported to keratinocytes around the melanocytes through dendrites to form supranuclear melanin caps which protect skin from photoaging. Nonetheless, melanin is also a key factor for skin disorders including age spots, freckles, and malignant melanoma. Therefore, tyrosinase inhibition is an effective approach to prevent excessive pigment deposition. Kojic acid, hydroquinone, and arbutin are commonly used in the treatment of melanin dermatosis due to their strong tyrosinase inhibitory effect. However, they are limited because of poor penetration and potential mutagenicity [26]. The present study revealed that the methanol extracts showed the highest tyrosinase inhibition among all the solvents tested (Stem: 82.93 ± 0.37 mg KAE/g; Fruit: 62.38 ± 0.55 mg KAE/g; Leaf: 69.92 ± 1.88 mg KAE/g). The high anti-tyrosinase activity of the methanol extract can be explained by the high TPC and TFC observed in the present study. Indeed, the positive correlation between TPC/TFC and tyrosinase inhibition was evidenced by Yang et al. [27].

One important therapeutic approach for the control of postprandial hyperglycemia in type 2 diabetes is through the inhibition of the digestion of dietary carbohydrates. Pancreatic α-amylase is an enzyme which breaks down dietary carbohydrates such as starch into simple monosaccharides followed by further degradation by α-glucosidases to glucose which is then absorbed and enters the bloodstream. Consequently, the inhibition of α-amylase and α-glucosidase can suppress carbohydrate digestion, delay glucose uptake, and, hence, lower blood glucose levels. Although some drugs such as acarbose, voglibose, and miglitol have been found to inhibit α-glucosidase and α-amylase, in practice, they cause some undesired adverse effects such as bloating, abdominal discomfort, diarrhea, and flatulence [28].

## 4. Conclusions

This study gives an insight on the biological effects and phytochemical profile of the various solvent extracts of the stem, fruit, and leaf of *P. spina-christi*. The methanol extract was found to be the most effective antioxidant and tyrosinase inhibitor and also possessed greater phenolic contents compared to other solvent extracts. Comparison of the parts revealed that the stem extract had higher TPC and antioxidant properties while the enzyme inhibitory potentials of the three parts were quite similar. This work provides a scientific basis for the potential use of *P. spina-christi* as a source of bioactive compounds for pharmaceutical drug development. Nonetheless, more in vitro, in vivo, and clinical studies need to be carried out, as well as determination of the bioavailability and toxicity profile of the species.

## Figures and Tables

**Table 1:** Annotated compounds in methanol and water Paliurus extracts from stems, fruits and leaves by LC-MS.

*m*/*z*	RT	Proposed Compound	Molecular Formula	Stem MeOH	Stem Water	Fruits MeOH	Fruits Water	Leaves MeOH	Leaves Water
341.1088	0.66	Sucrose	C12H22O11	+	+	+	+	+	+
195.0511	0.67	Gluconic acid	C6H12O7	-	-	-	+	-	-
503.1616	0.67	Raffinose	C18H32O16	-	-	+	+	-	-
609.1234	0.69	(Epi)gallocatechin-(epi)gallocatechin isomer	C30H26O14	+	-	-	-	-	-
593.1291	0.70	(Epi)gallocatechin-(epi)catechin isomer	C30H26O13	+	-	-	-	-	-
165.0408	0.71	Ribonic acid	C5H10O6	-	+	-	-	+	+
191.0563	0.72	Quinic acid	C7H12O6	+	+	+	+	+	+
305.0662	0.72	(Epi)gallocatechin isomer	C15H14O7	+	-	-	-	+	-
267.0721	0.77	Glucuronosylglycerol	C9H16O9	-	-	-	-	+	+
341.1083	0.83	Sucrose	C12H22O11	+	-	-	-	-	-
337.0768	0.89	Ascorbyl glucoside	C12H18O11	-	-	-	-	+	-
331.1034	1.57	Leonuriside	C14H20O9	-	+	-	-	-	-
563.1608	1.84	Pinocembrin rhamnosylglucoside	C23H32O16	-	+	-	-	-	-
401.1065	1.89	Apodanthoside	C17H22O11	-	-	-	-	-	+
315.0724	2.83	Protocatechuoylglucose	C13H16O9	-	+	-	-	-	+
609.1242	2.98	(Epi)gallocatechin-(epi)gallocatechin isomer	C30H26O14	-	+	-	-	-	-
401.1087	3.09	Apodanthoside	C17H22O11	-	-	-	-	-	+
467.1187	3.25	Catechin-ol galactopyranoside	C21H24O12	-	+	-	-	-	-
315.0718	3.69	Protocatechuoylglucose	C13H16O9	-	+	-	-	-	-
167.0350	3.72	Homogentisic acid	C8H8O4	-	+	-	-	-	-
303.0513	3.79	Taxifolin	C15H12O7	-	+	-	-	-	-
305.0670	3.80	(Epi)gallocatechin isomer	C15H14O7	-	+	-	-	+	+
331.0670	3.87	Glucogallin	C13H16O10	-	+	-	-	-	-
423.0718	4.06	Aurintricarboxylic acid	C22H16O9	-	+	-	-	-	-
305.0668	4.07	(Epi)gallocatechin isomer	C15H14O7	-	+	-	-	-	-
609.1242	4.08	(Epi)gallocatechin-(epi)gallocatechin isomer	C30H26O14	-	+	-	-	-	-
441.0821	4.09	(Epi)catechingallate	C22H18O10	+	+	-	-	-	-
423.0721	4.43	Aurintricarboxylic acid	C22H16O9	-	+	-	-	-	-
609.1249	4.44	(Epi)gallocatechin-(epi)gallocatechin isomer	C30H26O14	-	+	-	-	-	-
913.1809	4.49	(Epi)gallocatechin trimer isomer	C45H38O21	+	+	-	-	-	-
359.0984	4.62	Glucosyringic acid	C15H20O10	-	+	-	-	-	-
197.0459	4.62	Syringic acid	C9H10O5	-	+	-	-	-	-
319.0830	4.68	Methyl(epi)gallocatechin	C16H16O7	-	+	-	-	-	-
481.1355	4.68	Quercetin di-benzyl ether	C29H22O7	-	+	-	-	-	-
163.0407	4.96	Coumarinic acid	C9H8O3	-	-	-	-	-	+
401.1087	5.12	Apodanthoside	C17H22O11	-	+	-	-	-	+
913.1821	5.14	(Epi)gallocatechin trimer	C45H38O21	-	+	-	-	-	-
467.1177	5.17	Catechin-ol galactopyranoside	C21H24O12	-	+	-	-	-	-
593.1301	5.21	(Epi)gallocatechin-(epi)catechin isomer	C30H26O13	-	+	-	-	-	-
403.1970	5.25	Gynostemmoside A	C19H32O9	-	-	-	-	-	+
305.0669	5.33	(Epi)gallocatechin isomer	C15H14O7	+	+	-	-	+	-
565.2493	5.34	Euphorbioside A	C25H42O14	-	-	-	-	-	+
609.1244	5.46	(Epi)gallocatechin-(epi)gallocatechin isomer	C30H26O14	+	+	-	-	-	-
289.0719	5.63	(Epi)catechin	C15H14O6	-	+	+	-	+	+
913.1812	5.68	(Epi)gallocatechin trimer isomer	C45H38O21	+	+	-	-	-	-
535.2395	5.72	Octoacetylsaccharose	C24H40O13	-	-	-	-	+	+
549.2552	5.90	Nicoblumin	C25H42O13	-	-	-	-	+	+
425.0867	6.08	Sucrose Tricarboxylate Trimethyl Ester	C15H22O14	-	+	-	-	-	-
593.1285	6.10	(Epi)gallocatechin-(epi)catechin isomer	C30H26O13	-	+	-	-	-	-
441.0820	6.14	(Epi)catechingallate	C22H18O10	+	-	-	-	-	-
351.0722	6.15	Chlorogenoquinone	C16H16O9	-	-	-	-	+	+
595.1653	6.21	Eriodictyol neohesperidoside	C27H32O15	+	+	-	+	+	+
577.1346	6.21	Procyanidin B isomer	C30H26O12	+	+	+	-	-	-
897.1852	6.53	(Epi)gallocatechin (epi)catechin	C45H38O20	+	+	-	-	-	-
771.1968	6.53	Kaempferol sophorotrioside isomer	C33H40O21	-	-	-	-	-	+
405.2127	6.62	Euphorbioside B	C19H34O9	-	-	-	-	+	+
289.0716	6.63	(Epi)catechin	C15H14O6	-	+	-	-	-	-
449.1083	6.72	Astilbin	C21H22O11	+	+	-	-	+	+
287.0563	6.74	Dihydrokaempferol	C15H12O6	-	+	-	-	-	-
249.0619	6.85	Pyruvylmannose	C9H14O8	-	-	-	-	+	+
371.0982	6.86	Syringoylquinic acid	C16H20O10	-	+	-	-	+	+
577.1348	6.90	Procyanidin B isomer	C30H26O12	+	+	-	-	-	-
337.0929	7.04	Coumaroylquinic acid	C16H18O8	-	-	-	-	-	+
593.1499	7.20	Kaempferol rutinoside	C27H30O15	+	+	-	+	+	+
385.1135	7.25	Sinapoylglucose	C17H22O10	-	+	-	-	-	+
549.2543	7.33	Nicoblumin	C25H42O13	-	-	-	-	+	+
337.0925	7.45	Coumaroylquinic acid	C16H18O8	-	-	-	-	-	+
771.1966	7.54	Kaempferol sophorotrioside isomer	C33H40O21	-	-	-	-	-	+
887.2440	7.56	Quercetin xylosylrutinoside glucoside	C38H48O24	+	+	-	+	+	+
533.2596	7.57	Staphylionoside G	C25H42O12	-	-	-	-	-	+
337.0923	7.61	Coumaroylquinic acid	C16H18O8	-	-	-	-	+	+
741.1863	7.71	Quercetin xylopyranosylrutinoside	C32H38O20	+	+	+	+	+	+
771.1956	7.93	Kaempferol sophorotrioside isomer	C33H40O21	-	-	-	-	-	+
917.2553	7.97	Kaempferol rutinoside sophoroside isomer	C39H50O25	+	+	-	-	+	+
533.2594	8.01	Staphylionoside G	C25H42O12	-	-	-	-	+	+
609.1445	8.07	Quercetin glucoside	C27H30O16	-	-	-	-	-	+
581.2224	8.08	Manglieside E	C28H38O13	-	-	-	-	-	+
757.1808	8.12	Quercetin sambubioside glucoside isomer	C32H38O21	+	+	-	-	-	+
757.2068	8.14	Quercetin sambubioside glucoside isomer	C32H38O21	-	-	-	-	-	+
581.2224	8.18	Manglieside E	C28H38O13	-	+	-	-	-	+
607.1288	8.20	(Epi)catechin methyl(epi)gallocatechin	C31H28O13	-	-	-	-	-	+
755.2010	8.23	Quercetin rhamninoside	C33H40O20	+	+	+	+	-	+
609.1444	8.24	Quercetin glucoside isomer	C27H30O16	+	+	-	-	+	-
583.2298	8.28	Iryantherin C	C35H36O8	-	+	-	-	-	-
551.2123	8.30	Lyoniside isomer	C27H36O12	-	+	-	-	-	-
609.1435	8.32	Quercetin glucoside isomer	C27H30O16	-	+	-	-	-	+
243.1604	8.34	Tridecanedioic acid	C13H24O4	-	-	-	-	+	+
447.2226	8.36	Atractyloside A	C21H36O10	-	-	-	-	-	+
611.1239	8.40	Myricetin sambubioside	C26H28O17	+	+	-	-	+	+
609.1426	8.41	Quercetin glucoside isomer	C27H30O16	+	-	+	-	-	-
551.2126	8.62	Lyoniside isomer	C27H36O12	-	+	-	-	-	-
739.2066	8.69	Robinin	C33H40O19	-	-	-	-	+	+
607.1655	8.69	Diosmin	C28H32O15	-	-	-	+	-	-
593.1501	8.72	Kaempferol glucorhamnoside	C27H30O15	-	-	-	-	+	+
625.1397	8.74	Quercetin glucoside derivative	C27H30O17	-	-	-	-	+	+
583.2390	8.83	Yuanhuagine	C32H40O10	+	+	-	-	-	-
257.1183	8.90	Unknown	C16H18O3	+	+	-	-	+	+
449.2019	8.92	Hexaethylene glycol bis(acetoacetate)	C20H34O11	-	+	-	-	-	-
743.1909	8.95	Quercetin Xylopyranosyl Rutinoside	C32H40O20	-	-	-	-	+	+
739.2054	8.97	Robinin	C33H40O19	-	-	-	-	+	+
593.1488	9.00	Kaempferol rutinoside	C27H30O15	-	-	-	-	-	+
741.1862	9.01	Quercetin xylopyranosylrutinoside	C32H38O20	+	+	+	+	+	+
607.1657	9.23	Diosmin	C28H32O15	-	-	+	+	-	-
593.1135	9.36	(Epi)gallocatechin-(epi)catechin isomer	C30H26O13	-	-	-	+	+	+
301.0347	9.40	Quercetin	C15H10O7	-	-	+	-	-	-
595.1290	9.43	Quercetin vicianoside	C26H28O16	+	+	+	+	+	+
593.1135	9.52	(Epi)gallocatechin-(epi)catechin isomer	C30H26O13	-	-	+	-	-	-
641.1702	9.55	Haemocorin	C32H34O14	-	+	-	-	-	-
609.1442	9.61	Quercetin glucoside isomer	C27H30O16	-	-	-	+	-	+
607.1283	9.65	(Epi)catechin methyl(epi)gallocatechin isomer	C31H28O13	-	-	-	-	-	+
609.1442	9.75	Quercetin glucoside isomer	C27H30O16	+	+	+	+	-	+
607.1279	9.84	(Epi)catechin methyl(epi)gallocatechin isomer	C31H28O13	-	-	+	-	-	+
609.1439	9.91	Quercetin glucoside isomer	C27H30O16	+	+	+	-	-	-
463.0870	9.94	Isoquercitrin isomer	C21H20O12	-	-	-	+	+	+
461.0719	9.95	Luteolin glucuronide isomer	C21H18O12	-	-	-	-	+	+
461.0716	10.07	Luteolin glucuronide isomer	C21H18O12	-	-	+	-	+	+
463.0870	10.08	Isoquercitrin isomer	C21H20O12	+	+	+	+	+	+
299.0191	10.12	Dihydroxybutanedioic acid	C8H12O12	-	-	+	-	-	-
593.1498	10.19	Kaempferol rutinoside	C27H30O15	-	-	-	-	-	+
519.2798	10.21	Sedumoside G	C25H44O11	-	-	-	-	+	+
579.1343	10.23	Gambiriin A1	C30H28O12	-	-	-	-	+	+
225.1504	10.48	Methyl dihydrojasmonate	C13H22O3	-	-	-	-	-	+
243.1605	10.48	Tridecanedioic acid	C13H24O4	-	-	-	-	-	+
285.1712	10.48	Dimethyl epoxytridecanedioate	C15H26O5	-	-	-	-	-	+
917.2316	10.54	Kaempferol rutinoside sophoroside isomer	C39H50O25	-	-	-	-	+	+
593.1501	10.57	Kaempferol glucorhamnoside	C27H30O15	-	-	-	-	+	+
753.2008	11.25	Spinosin C	C37H38O17	-	-	-	+	-	-
901.2376	11.28	Kaempferol neohesperidoside coumarylglucoside isomer	C42H46O22	-	-	-	-	+	+
931.2485	11.37	Kaempferol neohesperidoside ferulylglucoside isomer	C43H48O23	-	-	-	-	+	+
885.2425	12.00	Kaempferol coumarylrobinobioside rhamnoside	C42H46O21	-	-	-	-	+	-
447.0930	12.25	Kaempferol glucoside	C21H20O11	-	-	+	-	-	-
901.2387	12.37	Kaempferol neohesperidoside coumarylglucoside isomer	C42H46O22	-	-	-	-	+	+
931.2490	12.42	Kaempferol neohesperidoside ferulylglucoside isomer	C43H48O23	-	-	-	-	+	+
755.1811	12.49	Scoparin Heptaacetate isomer	C36H36O18	-	-	-	-	+	-
755.1810	13.65	Scoparin Heptaacetate isomer	C36H36O18	-	-	-	-	+	-
301.0344	13.77	Flavonoid isomer	C15H10O7	-	-	+	-	-	-
327.2176	13.80	Malyngic acid	C18H32O5	+	+	-	-	+	+
183.1399	13.96	Undecanedial	C11H20O2	-	-	-	-	+	+
329.2340	14.40	Pinelli acid isomer	C18H34O5	-	-	-	+	-	-
329.2338	14.56	Pinelli acid isomer	C18H34O5	+	+	+	-	-	-
503.3369	15.25	Madecassic acid	C30H48O6	+	-	-	-	-	-
329.2338	15.53	Pinelli acid	C18H34O5	+	+	+	-	-	-
647.3803	15.71	Tragopogonsaponin A	C36H56O10	+	-	-	-	-	-
473.3267	16.34	Messagenic acid I	C29H46O5	-	-	+	-	+	-
315.1965	16.73	Cleistanthol	C20H28O3	+	-	-	-	-	-

+ present; - absent.

**Table 2:** Antioxidant properties of the tested extracts.

Parts	Solvents	DPPH (mg TE/g)	ABTS (mg TE/g)	CUPRAC (mg TE/g)	FRAP (mg TE/g)	MCA (mg EDTAE/g)	PBD (mmol TE/g)
Stems	n-hexane	11.05 ± 2.02 ^e^	48.61 ± 2.37 ^e^	60.17 ± 0.43 ^e^	52.93 ± 1.27 ^d^	9.45 ± 0.89 ^b^	1.66 ± 0.09 ^d^
	Ethyl acetate	90.82 ± 0.67 ^c^	394.24 ± 4.10 ^c^	180.42 ± 4.23 ^c^	149.78 ± 10.97 ^c^	10.28 ± 0.68 ^b^	2.12 ± 0.14 ^c^
	Dichloromethane	21.16 ± 2.55 ^d^	107.76 ± 0.97 ^d^	79.20 ± 2.91 ^d^	52.75 ± 1.43 ^d^	28.80 ± 0.32 ^a^	1.56 ± 0.07 ^d^
	Methanol	909.88 ± 4.25 ^a^	3358.33 ± 51.14 ^a^	781.88 ± 16.37 ^a^	996.70 ± 47.28 ^a^	6.15 ± 0.53 ^c^	4.96 ± 0.26 ^a^
	Water	547.54 ± 25.39 ^b^	1926.18 ± 34.63 ^b^	506.98 ± 2.54 ^b^	688.38 ± 3.43 ^b^	9.65 ± 0.35 ^b^	2.73 ± 0.05 ^b^
Fruits	n-hexane	1.30 ± 0.14 ^e^	12.43 ± 1.38	39.76 ± 1.01 ^d^	34.33 ± 1.78 ^d^	12.79 ± 1.33 ^b^	1.12 ± 0.04 ^c^
	Ethyl acetate	4.75 ± 0.22 ^d^	13.73 ± 0.61	45.12 ± 0.64 ^c^	45.19 ± 1.30 ^c^	7.31 ± 0.23 ^c^	1.07 ± 0.07 ^cd^
	Dichloromethane	5.44 ± 1.19 ^c^	16.88 ± 4.27	44.08 ± 2.62 ^c^	30.70 ± 1.42 ^d^	7.97 ± 0.37 ^c^	0.95 ± 0.07 ^d^
	Methanol	245.59 ± 4.46 ^a^	824.40 ± 17.11	282.66 ± 11.38 ^a^	292.94 ± 6.60 ^a^	8.53 ± 0.43 ^c^	1.63 ± 0.07 ^b^
	Water	161.23 ± 8.19 ^b^	694.79 ± 4.46	272.07 ± 6.41 ^b^	263.60 ± 1.89 ^b^	21.80 ± 0.59 ^a^	1.80 ± 0.03 ^a^
Leaves	n-hexane	16.43 ± 2.59 ^c^	51.83 ± 4.18 ^d^	55.48 ± 3.12 ^d^	48.48 ± 1.28 ^c^	22.31 ± 1.93 ^ab^	2.57 ± 0.13 ^a^
	Ethyl acetate	20.20 ± 1.31 ^c^	71.78 ± 1.77 ^c^	71.76 ± 1.82 ^c^	52.14 ± 0.23 ^c^	20.44 ± 0.38 ^b^	2.68 ± 0.17 ^a^
	Dichloromethane	4.88 ± 0.35 ^d^	45.60 ± 3.84 ^d^	73.45 ± 1.16 ^c^	54.82 ± 0.77 ^c^	23.44 ± 0.03 ^a^	2.58 ± 0.21 ^a^
	Methanol	480.10 ± 7.80 ^a^	1171.58 ± 25.83 ^a^	506.98 ± 7.27 ^a^	664.85 ± 0.49 ^a^	6.44 ± 0.28 ^c^	2.76 ± 0.19 ^a^
	Water	193.39 ± 2.50 ^b^	638.33 ± 14.11 ^b^	293.80 ± 10.03 ^b^	327.71 ± 5.89 ^b^	19.95 ± 0.25 ^b^	1.49 ± 0.05 ^b^

Values are reported as mean ± SD of three parallel measurements. TE: Trolox equivalent; EDTAE: EDTA equivalent. Different letters indicate significant differences in the extracts from same parts (*p* < 0.05).

**Table 3:** Enzyme inhibitory properties of the tested extracts.

Parts	Solvents	AChE (mg GALAE/g)	BChE (mg GALAE/g)	Tyrosinase (mg KAE/g)	Amylase (mmol ACAE/g)	Glucosidase (mmol ACAE/g)
Stems	n-hexane	8.89 ± 0.08 ^a^	1.95 ± 0.15 ^b^	40.91 ± 3.96 ^c^	0.49 ± 0.01 ^b^	1.01 ± 0.02 ^a^
	Ethyl acetate	8.28 ± 0.14 ^bc^	2.19 ± 0.15 ^b^	61.56 ± 2.29 ^b^	0.55 ± 0.01 ^a^	0.89 ± 0.04 ^a^
	Dichloromethane	8.17 ± 0.32 ^c^	2.16 ± 0.11 ^b^	39.01 ± 0.95 ^c^	0.55 ± 0.01 ^a^	1.07 ± 0.21 ^a^
	Methanol	8.64 ± 0.01 ^ab^	2.50 ± 0.05 ^a^	82.93 ± 0.37 ^a^	0.41 ± 0.01 ^c^	0.93 ± 0.06 ^a^
	Water	5.73 ± 0.03 ^d^	1.32 ± 0.05 ^c^	36.84 ± 0.20 ^c^	0.05 ± 0.01 ^d^	1.06 ± 0.01 ^a^
Fruits	n-hexane	8.50 ± 0.32 ^a^	2.18 ± 0.05 ^a^	35.08 ± 2.26 ^c^	0.52 ± 0.02 ^c^	na
	Ethyl acetate	8.07 ± 0.14 ^ab^	2.32 ± 0.13 ^a^	53.40 ± 5.84 ^b^	0.63 ± 0.01 ^a^	0.84 ± 0.07 ^a^
	Dichloromethane	8.37 ± 0.60 ^a^	2.32 ± 0.10 ^a^	35.12 ± 1.26 ^c^	0.55 ± 0.01 ^b^	0.83 ± 0.12 ^a^
	Methanol	7.26 ± 0.16 ^b^	0.48 ± 0.11 ^b^	62.38 ± 0.55 ^a^	0.43 ± 0.01 ^d^	0.98 ± 0.02 ^a^
	Water	1.07 ± 0.18 ^c^	0.32 ± 0.02 ^b^	3.59 ± 0.41 ^d^	0.17 ± 0.01 ^e^	1.00 ± 0.01 ^a^
Leaves	n-hexane	7.28 ± 0.18 ^a^	1.46 ± 0.12 ^a^	45.12 ± 3.73 ^b^	0.59 ± 0.03 ^a^	0.90 ± 0.04 ^ab^
	Ethyl acetate	8.37 ± 0.37 ^a^	1.68 ± 0.34 ^a^	43.18 ± 1.24 ^b^	0.60 ± 0.01 ^a^	0.86 ± 0.03 ^ab^
	Dichloromethane	7.93 ± 0.89 ^a^	1.98 ± 0.10 ^a^	47.64 ± 0.81 ^b^	0.60 ± 0.01 ^a^	0.77 ± 0.05 ^b^
	Methanol	8.41 ± 0.30 ^a^	0.49 ± 0.09 ^b^	69.92 ± 1.88 ^a^	0.30 ± 0.01 ^b^	1.00 ± 0.14 ^a^
	Water	2.60 ± 0.04 ^b^	na	9.45 ± 1.04 ^c^	0.05 ± 0.01 ^c^	1.01 ± 0.02 ^a^

Values are reported as mean ± SD of three parallel measurements. GALAE: Galanthamine equivalent; KAE: Kojic acid equivalent; ACAE: Acarbose equivalent; na: not active. Different letters indicate significant differences in the extracts from same parts (*p* < 0.05).

## Data Availability

Not applicable.

## Supplementary Materials

The following supporting information can be downloaded at: https://www.mdpi.com/article/10.3390/antiox12020255/s1, Table S1.Total phenolic and flavonoid content of the tested extracts; Figure S1. Base peak chromatogram of (A) methanol, (B) water steam extracts, (C) methanol, (D) water fruit extracts, (E) methanol and (F) water leaf extracts from *Paliurus*.
