# Supplementary material for: Phytochemical Profile and Biological Activities of Different Extracts of Three Parts of Paliurus spina-christi: A Linkage between Structure and Ability
Source: Antioxidants (Basel). 2023 Jan 23;12(2):255. doi: 10.3390/antiox12020255 (PMC9952067; doi:10.3390/antiox12020255)
Supplement: Supplementary file 1 [file antioxidants-12-00255-s001.zip › antioxidants-2157998-supplementary.pdf]

Table S1. Total phenolic and flavonoid content of the tested extracts

Parts	Solvents	TPC (mg GAE/g)	TFC (mg RE/g)
Stems	n-hexane	22.36±0.26[e]	0.83±0.07[e]
	Ethyl acetate	55.19±1.29[c]	7.84±0.26[c]
	Dichloromethane	30.50±0.82[d]	8.56±0.15[b]
	Methanol	121.78±1.41[a]	11.25±0.26[a]
	Water	105.14±0.99[b]	5.32±0.08[d]
Fruits	n-hexane	16.86±0.25[c]	0.14±0.03[e]
	Ethyl acetate	17.36±0.32[c]	0.51±0.03[c]
	Dichloromethane	16.58±0.75[c]	0.34±0.02[d]
	Methanol	75.91±0.58[a]	17.55±0.09[a]
	Water	68.97±0.36[b]	8.84±0.39[b]
Leaves	n-hexane	23.21±0.28[cd]	8.43±0.03[e]
	Ethyl acetate	25.72±0.10[c]	11.68±0.47[d]
	Dichloromethane	21.77±0.04[d]	21.56±0.14[c]
	Methanol	94.64±2.12[a]	75.36±0.92[a]
	Water	62.55±0.28[b]	42.18±0.26[b]

Values are reported as mean ±SD of three parallel measurements. TPC: Total Phenolic Content; TFC: Total Flavonoid Content; GAE: Gallic acid equivalent; RE: Rutin equivalent. Different letters indicate significant differences in the extracts from same parts ($p<0.05$).

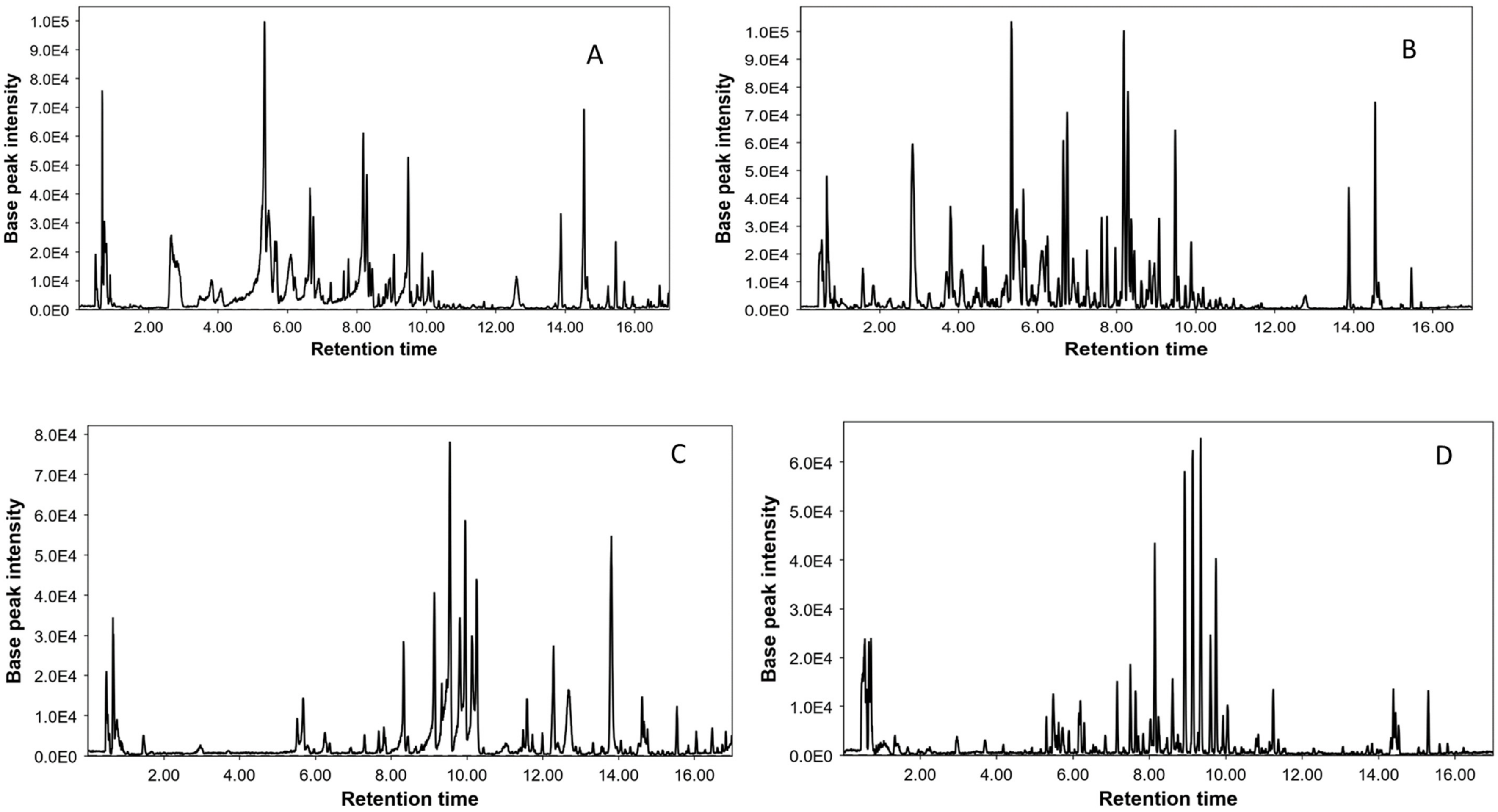
A
B
C
D
Base peak intensity
Retention time
0.0E0
1.0E4
2.0E4
3.0E4
4.0E4
5.0E4
6.0E4
7.0E4
8.0E4
9.0E4
1.0E5
2.00
4.00
6.00
8.00
10.00
12.00
14.00
16.00

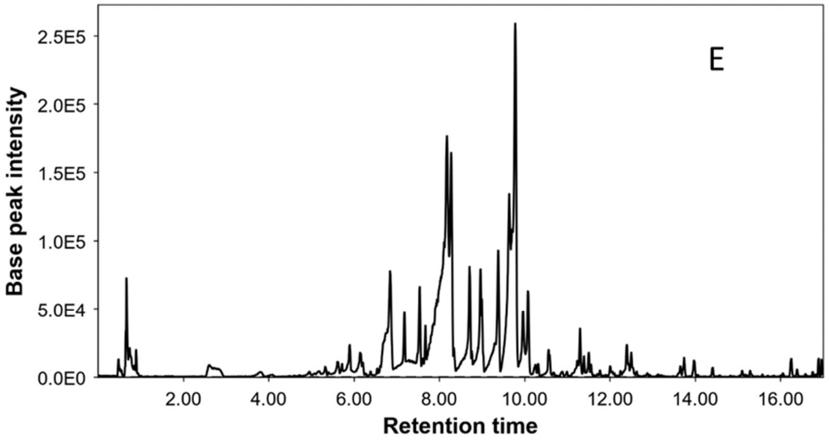

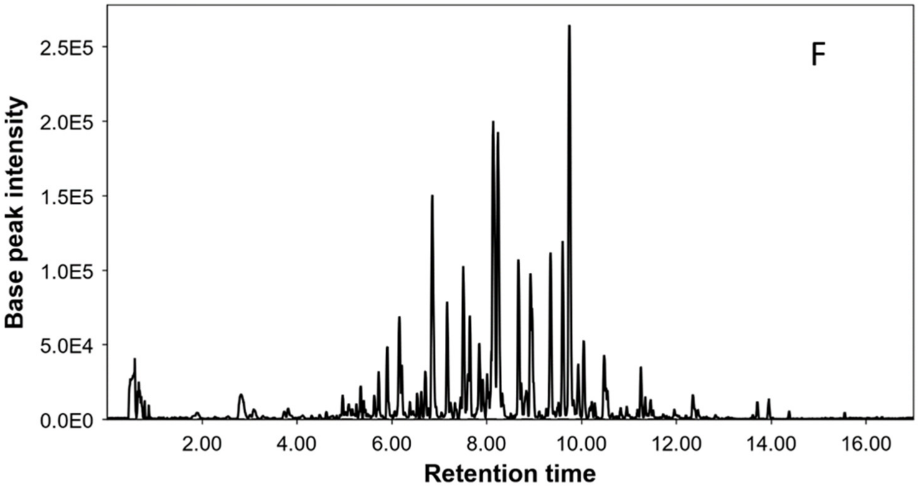

Figure S1. Base peak chromatogram of (A) methanol, (B) water steam extracts, (C) methanol, (D) water fruit extracts, (E) methanol and (F) water leaf extracts from *Paliurus*.
